# Elevated serum insulin‐like growth factor 1 in recurrent aphthous stomatitis

**DOI:** 10.1002/cre2.181

**Published:** 2019-03-27

**Authors:** Lorena Baccaglini, Jonathan J. Shuster, Douglas W. Theriaque, Zaeema Naveed

**Affiliations:** ^1^ Department of Epidemiology, College of Public Health University of Nebraska Medical Center Omaha Nebraska; ^2^ Health Outcomes and Policy, College of Medicine University of Florida Gainesville Florida; ^3^ College of Medicine University of Florida Gainesville Florida

**Keywords:** aphthous stomatitis, growth factors, IGF‐1, insulin, leptin

## Abstract

Over 100 million Americans experience recurrent aphthous stomatitis (RAS) at some point in life. To develop targeted drugs for RAS treatment, it is critical to identify its etiology. We determined if serum insulin‐like growth factor 1 (IGF‐1) and related factors are associated with RAS, because both RAS prevalence and IGF‐1 are highest during puberty. We analyzed data from 1,480 Third National Health and Nutrition Examination Survey participants aged 20–40 years. Participants with a history of diabetes or lupus, cotinine levels 6 ng/ml or higher or glycemia 110 mg/dl or higher were excluded. We compared levels of IGF‐1, IGFBP‐3, leptin, and insulin in participants with a positive vs. negative RAS history in the prior 12 months. We used logistic regression in SAS/SUDAAN to account for the complex sampling design. The odds of a positive RAS history were 1.31 times higher for every 100 ng/ml increase in serum IGF‐1. This association persisted after adjustment for age, race/ethnicity, medication intake, body mass index, insulin, leptin, glycemia, and income (adjusted OR = 1.30, 95% CI [1.06, 1.60]; *p* = 0.013). The odds of a positive RAS history were also higher among non‐Hispanic white compared with non‐Hispanic black participants (adjusted OR = 4.37, 95% CI [3.00, 6.38]). Leptin, IGFBP‐3, and insulin levels did not differ by RAS status. The significantly higher IGF‐1 levels in participants with a positive RAS history compared with controls suggest a possible role of the IGF‐1 pathway in RAS etiology.

## 1 INTRODUCTION

Recurrent aphthous stomatitis (RAS) is an inflammatory condition characterized by painful, recurrent ulcerations of the oral mucosa (Ship, Chavez, Doerr, Henson, & Sarmadi, 2000). Over 100 million individuals in the United States experience these painful ulcers at some point in life (Kleinman, Swango, & Pindborg, 1994). RAS is one of the most debilitating oral conditions (Robledo‐Sierra, Mattsson, Svedensten, & Jontell, 2013), due to the disproportionately high pain levels compared with the lesion size.

Different etiologic theories have been proposed over the years. There is general agreement that the immune system is activated in RAS, although there is no conclusive evidence that the initiating factor lies within the immune system. Furthermore, RAS is a very common condition affecting mostly healthy teenagers (Shulman, 2005). This unique age pattern is unlike most chronic autoimmune diseases that tend to be rare and life‐long.

Several treatments have been attempted through the years without strong evidence to support their use, aside from their nonspecific ability to reduce pain and inflammation. Patients find only temporary and often partial symptomatic relief from currently available medications, many of which have substantial side effects when used over prolonged periods of time to treat recalcitrant ulcers (Baccaglini et al., 2011; Thornhill, Baccaglini, Theaker, & Pemberton, 2007). Significant treatment progress can only be achieved through a better understanding of RAS etiology.

We had previously noted that insulin‐like growth factor 1 (IGF‐1) levels (Brabant et al., 2003) and RAS prevalence follow a remarkably parallel age curve, both reaching a peak during the growth spurt. Thus, we hypothesized that the specific pathway involving IGF‐1 might be the mechanism associated with wound healing and immune system activation in RAS patients.

IGF‐1 is a potent mitogen that enhances cell growth and proliferation and inhibits apoptosis (Duan, 2002; Lee, Kim, Kim, & Lee, 2010). The beneficial effects of IGF‐1 on maintaining the integrity of the gastrointestinal mucosa are well‐documented, although at high doses, IGF‐1 can induce aberrant healing (Coerper et al., 2001; Theiss, Fruchtman, & Lund, 2004). IGF‐1 is expressed by normal oral epithelial cells (Brady, Crean, Lorenzon, & Kapas, 2008) and is found in saliva (Antonelli, Gatti, Prearo, & De Palo, 2009). IGF‐1 receptors are widely distributed in the human body, including the oral cavity and salivary glands (Caviedes‐Bucheli et al., 2009; Katz et al., 2003). IGF‐1 binds primarily to the IGF‐1R receptor, and its actions are modulated by binding proteins (IGFBPs; Duan, 2002; Werner & Katz, 2004). Free IGF‐1 binds mostly to IGFBP‐3. During puberty, the increase in IGF‐1 is greater than that of IGFBP‐3, suggesting an increase in free, biologically active IGF‐1 (Juul et al., 1995). Intense cross‐talks exist between IGF‐1 and other growth factors, such as leptin and insulin. The insulin receptor (IR) can form a heterodimer with IGF‐1R and be activated by both IGF‐1 and insulin (Ryan & Goss, 2008). Both receptors also share IRS‐1/IRS‐2 and Shc as immediate downstream adaptors (Zielinski et al., 2009). Leptin levels are positively correlated with IGF‐1 and insulin (Woelfle, Harz, & Roth, 2007). Some of leptin's effects include: switching immune responses from Th2 to Th1 (Lord et al., 1998), stimulation of angiogenesis (Sierra‐Honigmann et al., 1998), and increased oral keratinocyte proliferation (Groschl et al., 2005). Leptin can also increase phosphorylation of the IGF‐1 signaling molecules IR substrate (IRS)‐1 and IRS‐2 (Saxena et al., 2008). Interestingly, smoking cessation, one of the recognized risk factor for RAS, increases leptin (Lee et al., 2006), whereas smoking is associated with lower IGF‐1 levels (Chelchowska et al., 2012) and lower RAS prevalence (Pentenero, Broccoletti, Carbone, Conrotto, & Gandolfo, 2008).

Thus, the aim of this study was to determine if RAS and IGF‐1, and IGF‐1‐related factors are associated in a nationally representative sample of the U.S. population. Our primary hypothesis was that participants with higher IGF‐1 serum levels have greater odds of having a positive RAS history compared with unaffected participants.

## 2 METHODS

We analyzed data from the Third National Health and Nutrition Examination Survey (NHANES Ill), a cross‐sectional survey conducted in the United States by the National Center for Health Statistics, Centers for Disease Control and Prevention (CDC). NHANES Ill is a nationally representative sample of the civilian noninstitutionalized U.S. population (1988–1994). The survey was approved by an Ethics Review Board, and all participants gave informed consent. Data were then collected using standardized questionnaires and examinations. A detailed description of the data collection methods is publicly available (CDC/NCHS, 1988).

Questionnaires were used to collect sociodemographic information such as age, gender, race/ethnicity, and tobacco use, medical history, and medication intake. Participants were also asked if they had canker sores or other ulcers or sores inside their mouth in the past 12 months, and this variable was used to classify patients as having a positive or negative RAS history. The examination component included oral and physical exams, blood draws, and other specific tests. Body mass index was calculated from height and weight measurements. Oral soft tissue examinations were performed by calibrated dentists to record existing lesions, including RAS, according to specified diagnostic criteria. Circulating biomarkers, such as insulin, cotinine and glucose, were measured through blood draws. Additionally, surplus sera from a subsample of participants aged 20 and older were analyzed for IGF‐1, IGFBP‐3, leptin, and other biomarkers.

All collected specimens were processed in designated laboratories using standardized procedures. A detailed description of specimen collection, processing, shipping, storage, and analytical procedures has been published (Gunter, Lewis, & Koncikowski, 1996).

Participants were included in our secondary analyses if they met certain eligibility criteria that had been established by CDC or reduced bias and variability (Table 1). For descriptive statistics, we calculated counts (%), means, and standard errors. We categorized continuous variables when appropriate using tertiles and clinically meaningful cutoffs. We calculated crude and adjusted OR and 95% confidence intervals (95% CI) for the association between RAS history and biomarker levels using logistic regression. For the initial data management, we used SAS software, version 9.3 (SAS Institute, Inc., Cary, NC). Then, to account for the multistage, clustered stratified sampling with oversampling and obtain population‐level results, we adjusted all estimates and standard errors using CDC's recommended cluster, strata, and weight (Mobile Examination Center (MEC) morning subsample weight) variables in SUDAAN 11.0.0. We selected a two‐sided α = 0.05 for all analyses.

**Table 1:** Eligibility criteria

NHANES III participant
Age 20–40 years
Surplus sera collected for IGF‐1, IGFBP‐3, and leptin after an overnight fast
Time of venipuncture before noon
No cigars, cigarettes, pipes, nicotine gum, chewing tobacco, or snuff in the past 5 days
Did not smoke cigarettes, eat a heavy meal, or use any medications to help his/her breathing 1 hr prior to venipuncture[Link]
Serum cotinine levels <6 ng/ml
Glycemia <110 mg/dl
Not diabetic (ever told by a doctor that had diabetes or taking insulin or oral hypoglycemic medications)
Never told by a doctor that had lupus
No missing values for major covariates

These three components were asked as a single question.

## 3 RESULTS

A total of 1,480 participants (293 with a positive and 1,187 with a negative RAS history) aged 20–40 years were eligible for inclusion in our analyses (Table 2). The age and gender distribution was similar for participants with and without RAS history in the prior 12 months. However, RAS history differed significantly by race (Tables 2, 3). Non‐Hispanic white participants had almost five times higher odds of reporting a positive RAS history compared to non‐Hispanic black participants.

**Table 2:** Participants' characteristics by RAS history; NHANES Ill (1988–1994)

Characteristic	Total		RAS history in past 12 months	
			Negative	Positive
	No. in sample	Estimated population (millions)	n (millions; %)	
Total	1,480	38.1	26.9 (70.6)	11.2 (29.4)
Gender				
Male	569	16.6	11.8 (71.1)	4.8 (28.9)
Female	911	21.5	15.1 (70.2)	6.4 (29.8)
Race/ethnicity				
Non‐Hispanic white	424	27.8	18.0 (64.8)	9.8 (35.2)
Non‐Hispanic black	448	4.5	4.0 (90.0)	0.4 (10.0)
Mexican–American	564	2.9	2.4 (83.6)	0.5 (16.4)
Other	62	2.9	2.4 (83.2)	0.5 (16.8)
Income^a^				
Less than $20,000	600	10.3	7.8 (75.7)	2.5 (24.3)
At least $20,000	860	27.5	18.8 (68.5)	8.7 (31.5)
Prescription medications in the past 24 hr				
No	1,208	29.5	21.6 (73.3)	7.9 (26.7)
Yes	272	8.6	5.3 (61.4)	3.3 (38.6)
Anti‐inflammatory medications in the past month				
No	776	16.8	12.6 (74.6)	4.3 (25.4)
Yes	704	21.3	14.3 (67.4)	6.9 (32.6)
Body mass index (kg/m^2^)				
Less than 24	579	16.9	11.3 (66.5)	5.7 (33.5)
24–28	441	11.8	8.4 (70.9)	3.4 (29.1)
Greater than 28	460	9.3	7.2 (77.6)	2.1 (22.4)
			Mean (SE)	
Age (years)^b^	1,480	38.1	30.5 (0.3)	30.2 (0.5)
IGF‐1 (ng/ml)	1,480	38.1	311.6 (5.1)	341.5 (7.9)
IGFBP‐3 (ng/ml)	1,480	38.1	4,756.8 (66.6)	4,844.0 (67.3)
Leptin (μg/ml)	1,480	38.1	10.7 (0.5)	9.5 (0.9)
Insulin (μIU/ml)	1,480	38.1	9.5 (0.3)	8.6 (0.5)
Glucose (mg/dl)^c^	1,480	38.1	89.1 (0.6)	88.2 (0.7)
BMI (Kg/m^2^)	1,480	38.1	25.9 (0.3)	24.9 (0.5)
Cotinine (ng/ml)^d^	1,480	38.1	0.4 (0.0)	0.4 (0.0)
Fasting (hr)	1,480	38.1	13.8 (0.1)	13.9 (0.1)

*Note*. RAS: recurrent aphthous stomatitis; SE: standard error; IGF‐1: insulin‐like growth factor 1; IGFBP‐3: insulin‐like growth factor binding protein 3. Estimates and SE are adjusted for the complex sampling design.

a Twenty observations were missing.

b Range 20–40.

c Maximum value is <110 mg/dl.

d Maximum value is <6 ng/ml.

**Table 3:** Crude and adjusted OR (95% Cl) comparing a positive vs. negative RAS history in the prior 12 months by selected participants' characteristics and growth factors; NHANES Ill (1988–1994)

Variable	No. in sample	Estimated population (millions)	Model 1 crude OR (95% CI)	Model 2 adjusted OR (95% CI)^a^	Model 3 adjusted OR (95% CI)^b^
Age (years)^c^	1,480	38.1	0.90 [0.66, 1.23]	0.99 [0.66, 1.48]	‐
Gender					
Male	569	16.6	1.00	‐	‐
Female	911	21.5	1.04 [0.71, 1.53]	‐	‐
Race/ethnicity					
Black non‐Hispanic	448	4.5	1.00^d^	1.00^d^	1.00^d^
White non‐Hispanic	424	27.8	4.89 [3.32, 7.22]	4.37 [3.00, 6.38]	4.82 [3.29, 7.04]
Other	608	5.8	1.79 [0.92, 3.46]	2.02 [0.96, 4.24]	2.05 [1.01, 4.14]
IGF‐1 (ng/ml)^e^	1,480	38.1	1.31 [1.12, 1.53]*	1.30 [1.06, 1.60]**	1.32 [1.12, 1.57]***
Prescription medications in the past 24 hr					
No	1,208	29.5	1.00****	1.00	1.00
Yes	272	8.6	1.73 [1.05, 2.83]	1.57 [0.88, 2.81]	1.60 [0.94, 2.76]
Anti‐inflammatory medications in the past month					
No	776	16.8	1.00*****	1.00	1.00
Yes	704	21.3	1.42 [1.01, 2.02]	1.21 [0.82, 1.79]	1.19 [0.81, 1.75]
Insulin (μIU/ml)^c^	1,480	38.1	0.67 [0.43, 1.04]	0.89 [0.59, 1.35]	‐
BMI (kg/m^2^)^c^	1,480	38.1	0.68 [0.45, 1.03]	0.89 [0.55, 1.44]	‐
Income^f^					
Less than $20,000	600	10.3	1.00	1.00	‐
At least $20,000	860	27.5	1.44 [0.94, 2.21]	1.07 [0.68, 1.69]	
Leptin (μg/ml)^c^	1,480	38.1	0.86 [0.66, 1.12]	0.98 [0.71, 1.37]	‐
Glucose (mg/dl)	1,480	38.1	0.99 [0.97, 1.01]	1.00 [0.98, 1.02]	‐
IGFBP‐3 (ng/ml)^e^	1,480	38.1	1.01 [0.99, 1.03]	‐	‐
Fasting (hr)	1,480	38.1	1.04 [0.92, 1.19]	‐	‐
Cotinine (ng/ml)	1,480	38.1	0.98 [0.76, 1.26]	‐	‐

*Note*. OR: odds ratio; Cl: confidence interval; IGF‐1: insulin‐like growth factor 1; BMI: body mass index; IGFBP‐3: insulin‐like growth factor binding protein 3. OR and 95% Cl are adjusted for the complex sampling design.

a Each variable is adjusted for all other variables in the model. Twenty (1.4%) observations were missing.

b Each variable is adjusted for all other variables in the model.

c 10‐unit increment.

d Overall *p* < 0.001.

e 100‐unit increment.

f 20 observations were missing.

* *p* = 0.001.

** *p* = 0.013.

*** *p* = 0.002.

**** *p* = 0.032.

***** *p* = 0.046.

Mean IGF‐1 levels were significantly higher in participants with a positive vs. negative RAS history (342 vs. 312 ng/ml). The crude odds of having a positive RAS history were 1.31 times higher for every 100 ng/ml increase in serum IGF‐1 (95% CI [1.12, 1.53]; Table 3, Model 1). This association persisted after adjustment for potential confounders (Table 3, Models 2 and 3).

Mean IGF‐1 levels were especially high in those individuals with a positive RAS history who also had recurrent aphthae upon exam (383 ng/ml; not shown in tables)^.^ However, this result should be interpreted with caution given the small number of individuals (*n* = 19).

Interestingly, although significantly fewer non‐Hispanic blacks reported a positive RAS history, their overall IGF‐1 levels (329 ng/ml) were higher than other races (non‐Hispanic whites: 324 ng/ml, Mexican–American: 285 ng/ml and other races: 307 ng/ml). However, among participants with a positive RAS history, non‐Hispanic blacks had higher IGF‐1 levels compared with all other races (380 vs. 340 ng/ml, respectively; not shown in tables).

A history of RAS was also more common in participants who had taken medications prior to the survey compared with those who had not, although the association was no longer significant after adjusting for other covariates (Table 2). Participants with a higher income, lower body mass index, and lower insulin levels had a greater, although not significant, tendency to report a positive history. However, these associations weakened considerably when other covariates, such as race/ethnicity and IGF‐1, were included in the model (Table 3, Model 2).

In summary, race and serum IGF‐1 were significantly and consistently associated with RAS history in the prior 12 months. Nonblack participants and participants with high serum IGF‐1 levels reported more frequently a positive history of RAS compared with blacks or participants with lower IGF‐1 levels. These two factors were strongly and independently associated with RAS and were unaffected by other participants' characteristics.

## 4 DISCUSSION

Our working hypothesis, developed during a series of RAS studies conducted by our research team, was that RAS is likely a wound healing abnormality related to an alteration in circulating IGF‐1 levels (or a closely linked factor). As a first step to test this hypothesis, we determined if an association between IGF‐1 and RAS could be detected by analyzing data from a national survey (NHANES III). Although this type of cross‐sectional design cannot prove a causal link, it provides important initial clues as to whether an association between RAS and the IGF‐1 pathway might exist.

We minimized age effects by restricting our sample to those aged 20–40 years. We selected participants aged 20 years and older because IGF‐1 levels were only measured in these individuals. We restricted the upper age limit to 40 years because IGF‐1 levels and RAS prevalence are highest in younger adults. If we had included older participants, most of them would have reported a negative RAS history in the prior 12 months, even if they had suffered from RAS as young adults. From our prior validation studies, we also knew that self‐reported RAS history alone is fairly accurate for diagnosing RAS in young adults, even when a single question is used (Baccaglini, Theriaque, Shuster, Serrano, & Lalla, 2013), but the accuracy of self‐reported RAS history in older adults is unknown. In this study, although we could not exclude misclassification bias, that is, some participants reporting accidental bites (other intraoral ulcers in young adults are very rare) instead of RAS, the effect of this bias would have been toward the null (i.e., to reduce the strength of association between RAS and IGF‐1), given that a link between accidental cheek bites and IGF‐1 is implausible. Despite concerns about a potential dilution of effects, we found a significant association between IGF‐1 and RAS. In addition, because race has not been linked to accidental bites, whereas the association between race and RAS is well‐known, our finding of a significant association between race and our primary outcome indirectly suggests that most NHANES participants were indeed reporting RAS, not other common “sores.”

Specifically, we found a significant crude association between IGF‐1 and RAS, with higher IGF‐1 levels in individuals with a positive RAS history compared with those with a negative history. This association persisted after adjustment for potential confounders. In multivariable models, the only effect that other covariates had on the IGF‐1 results was to reduce the precision of the confidence intervals, as expected when nonconfounders are included in a model (Table 3).

The negligible gender differences in RAS prevalence are consistent with prior reports. Because gender was not associated with the outcome, we excluded this variable from the multivariable models. Interestingly, blacks reported a significantly lower RAS prevalence, although their overall IGF‐1 levels were higher than other races. However, blacks who had experienced RAS in the prior 12 months had substantially higher IGF‐1 levels compared with participants of other races with a positive RAS history. The presence of a protective genetic factor in blacks could explain the apparent paradox of a generally lower RAS prevalence despite the high IGF‐1 levels and the higher RAS prevalence in blacks only in association with very high IGF‐1 levels.

An advantage of this study is that many potential confounders were measured using both self‐reports and laboratory techniques (such as cotinine for smoking and glycemia for diabetes and fasting), increasing the accuracy of these measurements. A limitation of this study is that we could not directly link IGF‐1 levels to the time of clinical RAS onset, although participants with active aphthae upon exam had the highest mean IGF‐1 levels (interpreting with caution this result due to the small sample size). Due to the cross‐sectional nature of the study, we also could not determine if or how IGF‐1 levels change before and after the appearance of an ulcer, although total IGF‐1 does not typically undergo rapid fluctuations.

By using restrictions at the design stage, we reduced the effects of major potential confounders and effect modifiers, such as smoking and hyperglycemic states. Exclusion of a sample with a particular factor may be more effective in controlling the cascade of complex underlying biological interactions triggered by that single factor than using statistical modeling, because most factors involved in biological pathways are unmeasured.

In conclusion, higher IGF‐1 levels and nonblack race were independently associated with a positive RAS history. These findings, which confirm previously reported strong racial differences in RAS history and unveil novel significant differences in IGF‐1 serum levels, provide support to our initial hypothesis. A common genetic abnormality differentially distributed across different races (i.e., less prevalent in blacks) and involving the IGF‐1 pathway, coupled with high circulating IGF‐1 levels could explain the differences in reported RAS history noted in this study. Additional studies are needed to assess changes in IGF‐1 levels temporally linked to clinical RAS onset and identify possible genetic variations linked to the IGF‐1 pathway in individuals with and without RAS history. Future studies could include more convenient measurements of salivary IGF‐1 levels (Antonelli et al., 2009) to determine the role of this growth factor in RAS and possibly other oral wound healing disorders.

## FUNDING INFORMATION

This work was supported by NIH grants NIH/NIDCR R03DE019687 and NIH/NCATS 1UL1TR000064.

## CONFLICT OF INTEREST

The authors have no conflicts to disclose other than the funding source.
